# The range and nature of non-motor symptoms in drug-naive Parkinson’s disease patients: a state-of-the-art systematic review

**DOI:** 10.1038/npjparkd.2015.13

**Published:** 2015-07-09

**Authors:** Panagiotis Zis, Roberto Erro, Courtney C Walton, Anna Sauerbier, Kallol Ray Chaudhuri

**Affiliations:** 1 National Parkinson Foundation International Centre of Excellence, King’s College London and Kings College Hospital NHS Foundation Trust, London, UK; 2 Sobell Department of Motor Neuroscience and Movement Disorders, University College London Institute of Neurology, London, UK; 3 Dipartimento di Scienze Neurologiche e del Movimento, Università di Verona, Verona, Italy; 4 Parkinson’s Disease Research Clinic, Brain and Mind Research Institute, The University of Sydney, Sydney, NSW, Australia

## Abstract

Non-motor symptoms (NMS) are a key component of Parkinson’s disease (PD). A range of NMS, most notably impaired sense of smell, sleep dysfunction, and dysautonomia are present from the ‘pre-motor’ phase to the final palliative stage. Theories as to the pathogenesis of PD such as those proposed by Braak and others also support the occurrence of NMS in PD years before motor symptoms start. However, research addressing the range and nature of NMS in PD has been confounded by the fact that many NMS arise as part of drug-related side effects. Thus, drug-naive PD (DNPD) patients provide an ideal population to study the differences in the presentation of NMS. The aim of this paper is therefore to systematically review all the available studies of NMS in DNPD patients. We believe this is the first review of its kind. The current review confirms the increasing research being conducted into NMS in DNPD patients as well as the necessity for further investigation into less-studied NMS, such as pain. Moreover, the data confirms non-motor heterogeneity among PD patients, and, therefore, further research into the concept of non-motor subtyping is encouraged. The review suggests that the clinical assessment of NMS should be integral to any assessment of PD in clinical and research settings.

## Introduction

Non-motor symptoms (NMS) are a key component of Parkinson’s disease (PD)^1^ and a major determinant of quality of life and phenotypic expression.^1,2^ Indeed, there is growing evidence that the overall burden of NMS may have a greater impact on quality of life than motor symptoms, not just in advanced motor disease as commonly perceived but also in early motor PD.^2,3^

A number of NMS are present from the ‘pre-motor’ stage^4^ to the final palliative stages of PD.^5^ The involvement of multiple neurotransmitters from the onset of PD, extra-striatal dopaminergic pathways, as well as early involvement of brainstem and olfactory areas in PD further augment the importance of NMS in PD.^6^

However, the fact that a range of NMS may arise as part of drug-related side effects confounds this issue further.^3^ Thus, drug-naive PD (DNPD) patients are an ideal population to study to study the differences in presentation of NMS at the early, or even pre-motor stages of PD and some controlled studies have also reported on the burden of NMS in DNPD.^7,8^ Yet, it is difficult to obtain large case series of DNPD patients as often patients referred to PD specialist centers or clinical research-orientated neurology departments are already on anti-parkinsonian medication. Thus the aim of this review is to systematically review all the available studies of NMS in DNPD patients and we believe this is the first review of its kind.

## Materials and methods

### Literature search strategy

**Figure 1:**
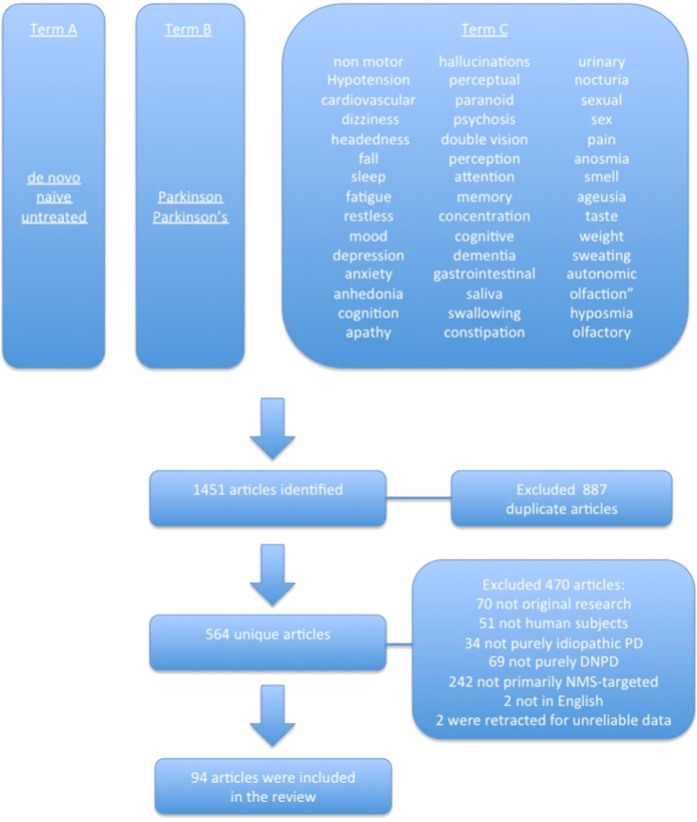
Prisma chart summarizing the literature search strategy and the application of inclusion and exclusion criteria.

A systematic literature search was carried out on the 4th of February 2015, using the PubMed database, covering all articles published up until that time. For each individual search, we used three Medical Subject Headings terms in either title or abstract (Figure 1). Term A was ‘*de novo*’, ‘naive’, or ‘untreated’; Term B was ‘Parkinson’ or ‘Parkinson’s’, and Term C was ‘non-motor’, ‘hypotension’, ‘cardiovascular’, ‘dizziness’, ‘headedness’, ‘fall’, ‘sleep’, ‘fatigue’, restless’, ‘mood’, ‘depression’, ‘anxiety’, ‘anhedonia’, ‘cognition’, ‘apathy’, ‘hallucinations’, ‘perceptual’, ‘paranoid’, psychosis’, ‘double vision’, ‘perception’, ‘attention’, ‘memory’, ‘concentration’, ‘cognitive’, ‘dementia’, ‘gastrointestinal’, ‘saliva’, ‘swallowing’, ‘constipation’, ‘urinary’, ‘nocturia’, ‘sexual’, ‘sex’, ‘pain’, ‘anosmia’, ‘smell’, ‘ageusia’, ‘taste’, ‘weight’, ‘sweating’, ‘autonomic’, ‘olfaction’, ‘hyposmia’, and ‘olfactory’.

The alternatives Medical Subject Headings for Term C were based on the Non-Motor Symptoms Scale (NMSS),^9,10^ and the Non-Motor Symptoms Questionnaire (NMSQuest).^11^ The NMSS is a 30-item clinician-rated scale designed to assess NMS in PD. The 30 items are grouped into nine domains: cardiovascular (2 items), sleep/fatigue (4 items), mood/cognition (6 items), perceptual problems (3 items), attention/memory (3 items), gastrointestinal (3 items), urinary (3 items), sexual function (2 items), and miscellaneous (4 items: pain, change in ability to taste or smell, change in weight, excessive sweating).^9,10^ The NMSQuest consists of nine NMS domains (gastrointestinal, urinary, attention/memory, perceptual problems/hallucination, sexual dysfunction, cardiovascular, sleep, mood/apathy, miscellaneous), each of which includes two to seven specific questions with dichotomous (yes/no) answers for a total of 30 items.^11^

We also perused the reference lists of the papers since the drafting of this paper so as to try and include further papers reporting aspects of NMS in drug-naive PD populations.

### Inclusion and exclusion criteria

To be included in the review, the articles had to meet the following inclusion criteria:

1. to be original papers,
2. to study human subjects,
3. to refer to studies which clearly address idiopathic PD only,
4. to refer to a strictly drug-naive population. As such, we have excluded studies where patients might have had a trial dose of any anti-parkinsonian agent and,
5. no retrospective studies as the study’s aim was to clinically explore the range and nature of NMS in prospective study-based papers.

## Results

### Search results

The search strategy described above resulted in the identification of 1,451 articles. Out of these, 887 articles were duplicates as different combinations of Terms A, B, and C resulted in the same article in many cases. After the eligibility assessment, 468 articles were further excluded, as they did not meet the inclusion criteria. Two papers were further excluded as their publication have been retracted for unreliable data. In total, 94 papers were included in the review. Table 1 summarizes the characteristics of these papers. Figure 1 illustrates the study selection process.

### Holistic NMS profile of drug-naive PD patients

The majority of studies that were designed to describe the holistic NMS profile of drug-naive PD patients used the NMSS and the NMSQuest.

In 2009, Kim *et al.*^12^ were the first to use the NMSS to describe the holistic NMS profile of DNPD patients compared with controls. NMS were significantly more frequently present in the DNPD population, with nocturia, forgetfulness, and restless legs being the three most common symptoms. Interestingly, neither age nor duration of disease was correlated with the number of NMS or the total NMSS score. However, a positive correlation between the number of NMS or the total NMSS score and motor disease severity as assessed by Hoehn and Yahr stage was observed.^12^

A major study of 1,072 patients in 2009 by Barone *et al.*,^13^ the PRIAMO study described the NMS profile of 107 DNPD patients via a semi-structured interview (the PRIAMO-Quest), which consisted of 12 NMS domains (gastrointestinal symptoms, pain, urinary symptoms, cardiovascular symptoms, sleep disorders, fatigue, apathy, attention, skin disorders, psychiatric symptoms, respiratory symptoms, other symptoms). The highest prevalence of NMS in DNPD patients was found to be psychiatric NMS, such as anxiety and depression (present in 66% of the DNPD population), followed by fatigue (present in 52% of the DNPD population). The least common NMS was found to be respiratory problems, such as stridor, cough, and dyspnea, (present in 8% of the DNPD population).

In 2011, Müller *et al.*^14^ studied the autonomic and sensory symptoms in 207 untreated PD patients using a preliminary version of the Movement Disorder Society-sponsored revised version of the UPDRS (pMDS-UPDRS). They found that both categories of symptoms were more frequent in patients compared with controls. In the patient group, reduced olfaction (59%), urinary problems (47%), increased saliva or drooling (42%), constipation (39%), and sensory complaints such as pain or abnormal sensation (34%) were the most frequent symptoms but mild in severity, although severity was not assessed by validated cut-off scores of specific non-motor instruments.^14^ Similar to the observation by Kim *et al.,*^12^ higher Hoehn and Yahr stage was associated with a larger number of autonomic and sensory symptoms and with the occurrence of gastrointestinal symptoms. However, a recent study by Zis *et al.*^3^ suggests that the burden of NMS in DNPD subjects can be severe (26.3%) and very severe (19.3%).

In 2013, an analysis of the baseline data of the DeNoPa cohort was performed and used both NMSQuest as well as NMSS in addition to SCOPA-AUT scale.^7^ The findings showed that DNPD patients, compared with controls, present with an increase in the total NMSQuest score, the total NMSS scores, and all SCOPA-AUT subscores (gastrointestinal, urinary, cardiovascular, thermoregulatory, pupilomotor, and sexual dysfunction in men) sparing sexual dysfunction in women. In a large DNPD population consisting of 200 patients, Picillo *et al.*^15^ found that using the NMSQuest, only 11.5% of patients were free of NMS, whereas 21.5% of healthy controls were free of NMS. All NMS domains were affected, with the exception of perceptual problems/hallucinations. The most prevalent symptoms were anxiety and sadness. Although overall NMSQuest scores did not differ between males and females, the former complained significantly more frequently of sexual problems and taste/smelling difficulties.

In 2013, Kim and colleagues^16^ compared NMSS symptoms across DNPD and drug-induced parkinsonism groups using the NMSS. Their results suggest that NMS were prevalent in the DNPD group, and symptoms of urinary and sleep disturbance, restless legs syndrome (RLS), attention deficits, and hyposmia were able to differentiate these groups, once controlling for age and gender.

Contrary to the gender findings above, in a similar study conducted by Song *et al.* in 2014, it was shown that DNPD female patients were more depressed and had more impaired cognition, whereas gender differences were not apparent on motor and other NMS.^17^ A holistic analysis of the NMS spectrum in the latter study population showed that 71% of the patients report fatigue, 59% of the patients were having sleep problems, 36% of the patients suffered from constipation, and 32% of the patients suffered from depression.^18^ However, in this study, the authors did not use a single holistic NMS tool, but other specific questionnaires such as the 20-item version of the Center for Epidemiological Studies Depression Scales (CES-D) for depression and the MMSE and Alzheimer’s Disease Assessment Scale-Cognitive (ADAS-Cog) for cognition.

Also in 2014, Zis *et al.*^3^ analyzed cross-sectional UK data from a multicenter collaboration, using the NMSS and showed that NMS are common in DNPD patients and over 45% may have severe to very severe burden of NMS, a key determinant of quality of life.

In a recent paper, Yang *et al.*^19^ compared the holistic NMS profile between DNPD patients and SWEDD (scans without evidence of dopaminergic deficits) patients. Using validated scales including the NMSS, they showed that DNPD patients have more NMS than newly diagnosed untreated PD patients, suggesting that specific NMS, especially rapid eye movement (REM) sleep behavior disorder (RBD) or olfactory impairment, might aid the differential diagnosis, regardless of what the actual condition underlying SWEDD is.^19^

The ONSET-PD study aimed to describe the presence and perceived onset of NMS in PD, using the NMSQuest and the SCOPA-aut.^20^ Anhedonia, apathy, memory complaints, and inattention were found to anticipate the motor onset and occurred more frequently in the DNPD patients compared with controls during a 2-year pre-motor period. Hyposmia, mood disturbances, ageusia, excessive sweating, fatigue, and pain were noted in the 2- to 10-year pre-motor period. Constipation, dream-enacting behavior, excessive daytime sleepiness, and postprandial fullness were frequently perceived more than 10 years before motor symptoms. These findings confirm that NMS are prevalent in early DNPD and frequently reported to occur in the pre-motor period.^20^

All these studies confirm that NMS are present in early untreated stages of PD and that differences between genders and among populations exist. Such differences in NMS profiling suggest that there is non-motor heterogeneity in PD right from the onset of the motor disorder. Thus, an interesting and elegant study, conducted by Erro *et al.*^21^ took this hypothesis further by using a data-driven approach on DNPD. They identified four distinct groups of patients, which they have labeled: (1) benign pure motor; (2) benign mixed motor-non-motor; (3) non-motor dominant; and (4) motor dominant, promoting the hypothesis that non-motor subtyping is possible. The ONSET-PD study also report specific non-motor clustering.^20^ Specific NMS-based studies (as reviewed below) support such a suggestion and therefore non-motor subtyping is of great interest and has indeed recently gained momentum.

### Specific NMS studies

#### Autonomic NMS

Features of autonomic disturbance include symptoms caused by sympathetic dysfunction, parasympathetic dysfunction, or both. These symptoms may concern, among others, cardiovascular symptoms, urinary symptoms, sexual dysfunction, and other issues such as excessive sweating.

#### Cardiovascular dysfunction

Untreated PD patients suffer significant failure in cardiovascular nervous system regulation.^22^ There is reasonable evidence to suggest that DNPD patients show sympathetic dysfunction,^22–29^ the most prominent symptom of which is orthostatic hypotension, which may lead to considerable morbidity. The prevalence of orthostatic hypotension varies from 4% (ref. 23) to 60%.^30^ In addition, Hiorth *et al.*^31^ observed that 17% of untreated patients may experience falls that could possibly be linked to orthostatic hypertension.

Many studies have aimed to investigate heart rate (HR) variability and resting HR in DNPD patients. Both the HR response to standing and the HR variability to deep breathing are tests used to assess the parasympathetic function. DNPD patients show significant impairments in HR variability^22–24,28,29,32,33^ compared with controls. Moreover, the DeNoPa study showed that mean HR is increased in DNPD patients, whereas the QT interval is shorter compared with healthy controls.^7^ However, data on parasympathetic dysfunction in early, untreated, stages of PD remains largely inconclusive. Although the majority of the studies assessing cardiovascular reflexes such as the Ewing’s battery of autonomic tests, showed that parasympathetic dysfunction may be present early in the disease course,^22–24,27–29,32,33^ some studies have shown that DNPD manifested only sympathetic dysfunction, either assessing cardiovascular reflexes or on the basis of cardiac radioiodinated metaiodobenzylguanidine uptake.^34^

PD causes dysfunction of the diurnal autonomic cardiovascular regulation.^32^ Moreover, during sleep, DNPD patients have been found to show defective cardiac autonomic control,^35–37^ mainly parasympathetic but also sympathetic in nature, despite having normal results in conventional autonomic tests during wakefulness.^35^

Postprandial hypotension is another cardiovascular symptom experienced in PD. As early as in 1987, Micieli *et al.*^30^ showed that over 50% of patients studied showed a marked postprandial systolic fall resembling that observed in chronic autonomic failure. In 2014, Umehara *et al.*^38^ concluded that in DNPD, systemic sympathetic denervation, impaired baroreflex-cardiovagal gain, and insufficiency of compensatory sympathetic nervous activation is associated with postprandial hypotension. Thus, systemic sympathetic denervation and baroreflex failure seem to contribute toward the development of postprandial and orthostatic hypotension.

#### Urinary dysfunction

Urinary NMS include difficulty holding urine (urgency), increased urinary frequency, and nocturia and may be the result of storage or voiding dysfunction. In 2011, Uchiyama *et al.*^39^ calculated that 64% of DNPD patients report urinary symptoms, while 82% have abnormal urodynamic studies. However, the ONSET-PD study^20^ showed that urinary symptoms are not more prevalent in DNPD patients compared with controls, at least in the early PD stage.

Interestingly, urinary symptoms were not found to correlate with gender, disease severity or motor symptom type.^20^ However, Erro *et al.*^40^ have recently showed that DNPD patients with urinary symptoms have higher motor and non-motor disturbances than those without suggesting the existence of a subgroup of patients who have an overall higher motor and non-motor burden and increased likelihood to require levodopa over the first 4 years from diagnosis. On the basis of these findings, Erro *et al.*^40^ argued that urinary symptoms might be a clinical marker of severity as well as possibly highlighting a non-motor subtype of PD.

#### Gastrointestinal dysfunction

Gastrointestinal symptoms in PD include dysphagia, nausea, constipation, and defecatory dysfunction.^41^ DNPD patients show delayed gastric emptying^42,43^ and reduced bowel sounds^44^ compared with healthy controls. Interestingly, Tanaka *et al.*^45^ took this further and showed that delay in gastric emptying did not differ between untreated, early-stage, and treated advanced-stage PD patients. In 2011, Tanaka *et al.*^45^ showed that gastric emptying of untreated, early-stage PD is already delayed, thus making delayed gastric emptying a potential marker of the preclinical stage of PD.

In 1991, Edwards *et al.*^41^ observed that gastrointestinal symptoms are comparable between treated and untreated PD except for defecatory dysfunction, which was significantly more common in treated patients and suggested that the gastrointestinal symptoms of PD reflect direct involvement in the gastrointestinal tract by the primary disease process. In 2011, almost 95% of the patients recruited in the QL-GAT study reported constipation, which was ameliorated after treatment with levodopa.^46^

#### Hypersialorrhea (drooling)

Hypersialorrhea is frequently reported in patients with PD and may be the result of excessive production of saliva, swallowing difficulties, or both. In a study designed to prospectively investigate the salivary secretion, Bagheri *et al.*^47^ found that DNPD patients show a lower salivary flow compared with controls, whereas no significant differences were seen between untreated and treated patients, suggesting that hypersialorrhea could better be explained on the basis of swallowing difficulties. On the other hand, decreased salivary flow might be explained by autonomic dysfunction.

#### Rhinorrhea

In a case–control study conducted through interviewing and using a specific questionnaire, it was estimated that half of the DNPD patients suffered from rhinorrhea.^48^ The authors hypothesize that the high frequency of rhinorrhea in PD patients may be due to sympathetic dysfunction. Moreover, the authors showed an association between rhinorrhea and olfactory dysfunction. Whether rhinorrhea may actually affect olfaction, as tested with objective measures, is yet to be determined.^48^

### Neuropsychiatric symptoms

A wide range of neuropsychiatric symptoms have been described in PD even in early and untreated patients.^49^ Only few studies have focused on the whole spectrum of neuropsychiatric symptoms in DNPD patients, the majority having investigated only specific symptoms. In 2012, Poletti *et al.*^50^ in a small series of DNPD patients administered a neuropsychiatric battery, finding that the most common neuropsychiatric symptoms included depression (33%), alexithymia (20%), anxiety (20%), and impulsivity (10%). In 2014, Aarsland *et al.*^49^ used the Neuropsychiatric Inventory in a large unselected sample of patients with incident, non-demented DNPD, and compared the results using age- and education-matched controls. More than half of the patients exhibited at least one symptom, and more than 25% had at least one symptom of clinically significant severity. Several patients had two or more symptoms, and 13% had two or more clinically significant symptoms. Depression, anxiety, sleep disturbances, and apathy were the most common symptoms, whereas psychotic symptoms were very rare.^49^

In a large controlled study on the global spectrum of neuropsychiatric symptoms, de la Riva *et al.*^51^ showed that depression, fatigue, apathy, and anxiety are significantly more prevalent in DNPD patients compared with healthy controls, despite their tendency to remain relatively stable in the early stage, whereas global cognition slightly deteriorates.

#### Depression

Depressive symptoms are common in DNPD patients, with a prevalence varying from study to study. Younger age of PD onset is associated with depression in DNPD patients^52^ and, interestingly, Santamaria *et al.*^52^ observed that in 91% of depressed DNPD patients, depressive symptoms began before the motor symptoms.

Ravina *et al.*^53^ pooled the baseline data from two phase II clinical trials enrolling early, untreated subjects with PD showing that out of a total of 413 patients, 13.8% suffered from depression. In smaller series, depressive symptoms have been shown to be present in approximately one-third of patients.^50–52,54^ Choi *et al.*^54^ showed that after long-term levodopa therapy, up to 18% of depressed patients may recover, whereas 22% of the initially nondepressed patients became depressed. In 2014, Spalletta *et al.*^55^ showed that following treatment, depressive symptoms improvement was paralleled by the very expected improvement of motor symptoms severity. However, change in depression severity over a long term was not significantly related to changes in motor symptoms.^55^

#### Anxiety

Studies focusing purely on anxiety in DNPD patients are limited. In a small case series, Spalletta *et al.*^55^ estimated that the frequency of generalized anxiety disorder was 8.3%, a percentage not altered following treatment.

#### Apathy

The prevalence of apathy within DNPD ranges from as low as 8.3% (ref. 55) to 33.3%.^56^ In a large controlled study, Pedersen *et al*.^57^ showed that apathy was significantly associated with male gender, higher depression scores, and more severe motor symptoms, but was not associated with greater cognitive impairment. However, Dujardin *et al.*^58^ in their DNPD population showed that apathy was associated with lower cognitive status, as well as with fatigue and anhedonia. A self-report version of the Apathy Evaluation Scale has now been validated, for detecting apathy in PD,^56^ and this tool may help to clarify these conflicting results in future studies.

#### Alexithymia

In a small controlled study, Poletti *et al.*^59^ estimated that 23.8% of the DNPD patients are alexithymic, 26.2% borderline alexithymic, and 50% non-alexithymic. These percentages were not statistically different compared with controls. On the other hand, they observed that DNPD with the postural instability gait difficulty motor subtype presented more alexithymic features than PD patients with tremor-dominant phenotype, suggesting that the postural instability gait difficulty subtype could represent a risk factor for developing alexithymia.^60^

#### Impulsivity—compulsivity

The association between impulse control disorders (ICDs), including compulsive gambling, buying, hypersexuality, binge eating, and punding, and dopaminergic medications is well established.^61^ Conversely, only few studies have investigated the frequency and correlates of impulse control and related behavior symptoms in DNPD patients. In 2011, Antonini *et al.*^62^ found that, despite none fulfilled DSM-IV criteria for any ICDs, 18 of 103 (17.5%) DNPD patients screened positive for at least one ICD using the Minnesota Impulsive Disorder Interview and the South Oaks Gambling Scale, a figure similar to that of healthy controls. Similarly, a large study conducted in 2013 by Weintraub *et al.*,^61^ estimated that at least one ICD was present in 18.5% of DNPD patients. Specifically, binge eating was the most frequent ICD (7.1%), followed by hobbyism (5.4%), punding (4.8%), hypersexuality (4.2%), buying (3.0%), gambling (1.2%), and walkabout (0.6%). In a study that included a small number of DNPD patients, Nicoletti *et al.*^63^ estimated that 50% of them presented with obsessive-compulsive personality disorder. These findings highlight the necessity of a detailed behavioral assessment before starting dopaminergic therapy.

#### Memory—cognition

Although dementia is a feature of advanced PD, mild cognitive impairment can occur in the early stages of PD.^64^ Several studies have been conducted and investigated the performance of DNPD patients across multiple neuropsychological tests.^55,65–74^ Memory impairment in early DNPD mainly reflects deficit of learning and encoding rather than retention or retrieval.^66^ This pattern of cognitive impairment is distinct from the one occurring in the normal ageing process.^65^ With medical treatment, selective improvement in memory domains (verbal and visuo-spatial episodic memory) has been observed.^55^

Compared with controls, DNPD patients have a relative risk ratio of 2.1 to develop mild cognitive impairment.^75^ The prevalence of mild cognitive impairment varies from study to study ranging from 14.8% (ref. 70) to 43.4% (ref. 76), and this may reflect ongoing uncertainty of the most appropriate diagnostic criteria.^77^ On the other hand, the presence of dementia seems also to depend on the time of PD onset and, therefore, often on the age of the patient. Reid *et al.*^65,78^ estimated that 39% of DNPD patients whose symptoms of PD began after the age of 70 years had dementia, while only 8% of DNPD patients whose symptoms of PD began before the age of 70 years did have dementia.

Another interesting association is the relationship between sleep and cognitive dysfunction.^79^ Specifically, both RBD and insomnia have been associated with lower scores on several cognitive tests. Given the correlation between sleep disturbances and cognitive impairment, it is possible that sleep symptoms in PD patients might be considered as an early marker of cognitive decline.^79^

In an interesting study published in 2014, Kwon *et al.*^80^ studied the whole NMS spectrum in 80 DNPD patients and suggested that depression, vivid dreaming, RBD, hyposmia, and abnormal stereopsis are closely associated with cognitive decline, and that presence of these NMS predicts the subsequent development of dementia. In a small series by Bae *et al*.,^81^ orthostatic hypertension was shown to be associated with cognitive dysfunction in DNPD patients.

### Fatigue

Fatigue is a common symptom in PD, since the earliest stage, and it has a negative impact on patients’ activities of daily living.^82^ Herlofson *et al.*^82^ estimated that 55% of the DNPD patients suffer from clinically significant fatigue, an amount almost three times greater than controls. Kang *et al*.^83^ estimated that 45% of the DNPD patients suffer from fatigue independently from their motor burden, further showing that depression and difficulties with activities of daily living were independent risk factors for fatigue. Conversely, the analysis of the baseline data of the ELLDOPA study showed that 37% of untreated PD patients suffer from fatigue.^84^ Fatigue was associated with the severity of PD, and progressed less in patients who subsequently were treated with levodopa.^85^

### Sleep

Apart from nocturia and autonomic dysregulation during sleep, which have been described above, other sleep disturbances may emerge from the early, untreated stages of PD.^85–88^

#### Periodic limb movements

In a polysomnographic study, Wetter *et al.*^89^ showed that sleep disruption and increased motor activity during REM and non-REM sleep are a frequent finding in DNPD patients. They also found an increased periodic limb movement index, which may be due to a dopaminergic deficit and is probably not associated with dopaminergic treatment.^89^ However, the same group did not confirm these results in a secondary study.^90^

#### Restless leg syndrome

The prevalence of restless leg syndrome (RLS) varies from 5.5% (ref. 91) to 16.5%.^92^ Angelini *et al.*^91^ showed that the frequency of overall life-time RLS did not differ significantly between DNPD patients and controls. In another study, Gjerstad *et al.* estimated that about 15.5% of DNPD patients meet RLS criteria, a percentage that was not significantly higher compared with controls.^93^ Nevertheless, they found that leg motor restlessness occurred with a near three-fold higher risk in PD patients as compared with controls.^93^ These conflicting findings underline the need for more accurate assessments of RLS in PD and support the notion that RLS and PD are different entities.^93^

#### REM sleep disorders

Video-polysomnographic studies have demonstrated that 28% of the DNPD patients show REM sleep without atonia, a percentage that is significantly higher compared with controls.^85^ In 2013, Plomhause *et al.*^94^ estimated that 30% of the DNPD patients met the criteria for RBD. In a larger video-polysomnographic study, Sixel-Döring *et al.*^95^ detected REM sleep behavioral events in 51% of the DNPD patients, a percentage significantly higher compared with controls. Similar to the study by Plomhause *et al.,*^94^ RBD was identified in 25% of the total DNPD population.^95^

#### Excessive daytime sleepiness

In 2002, in a case–control study, Fabbrini *et al.*^96^ showed that excessive daytime sleepiness does not seem to be a trait of untreated PD but appears only in treated PD patients. This was also shown by Kaynak *et al.* in 2005.^97^ However, more recently Giganti *et al.*^98^ showed a higher level of sleepiness in the DNPD patients compared with controls in the hours following awakening and in the early afternoon. Their results suggest that sleepiness during specific daytime may be an early manifestation of sleepiness, which will spread later to the whole daytime.^98^

### Sensory dysfunction

#### Olfactory dysfunction

Olfactory symptoms include reduced ability (hyposmia) or inability (anosmia) to smell. Decreased olfactory function is among the first signs of idiopathic PD and is probably present in the pre-motor phase.^99,100^ Apart from odor identification, odor discrimination is also affected in DNPD patients.^101^ The olfactory dysfunction is bilateral and not related to motor symptoms.^99^

#### Ophthalmologic dysfunction

A wide spectrum of ophthalmologic features may be present in DNPD patients. In a controlled study, Biousse *et al.*^102^ showed that ocular complaints are more common in DNPD compared with controls, with ocular surface irritation being the most common (63.3%), followed by difficulty in reading (26.7%), visual hallucinations (26.7%), and diplopia (10%). Two studies showed that DNPD patients show alterations in chromatic contour perception, which is correlated to the severity of the disease.^103,104^ Interestingly, up to 87.5% of DNPD patients were found to suffer from dysfunction of stereopsis and visual perception.^105^

## Conclusion

This systematic review aimed to focus on the NMS of DNPD patients. It is the first review on this topic and is of particular interest as it may not only offer a better insight on early symptoms that may contribute to the diagnosis, or be used as potential clinical markers, but also may provide a better understanding of which NMS are affected by dopaminergic treatment. Our review indicates the following key points:

1. Research in DNPD is increasing. This is exemplified in our data (Table 1), which shows that the number of papers reporting NMS in DNPD patients after 2011 has exceeded the total numbers of papers reporting NMS in DNPD patients up until 2010, a period spanning over 20 years.
2. While initial studies in DNPD were uncontrolled and usually relied, at one point in time, on cross-sectional design, many are now controlled. DNPD patients are often described as ‘gold dust’ as they are difficult to recruit into studies as often anti-parkinsonian medication would have been been started before their presentation to tertiary, specialist, PD centers. This highlights the necessity of a multicenter approach to studies related to DNPD. This review confirms that there is considerable burden of non-motor symptoms and heterogeneity in DNPD patients in addition to motor presentations.^21^ These observations underpin the concept for non-motor subtyping within PD as certain NMS are overrepresented in DNPD and are likely to be dominated by these NMS, such as sleep dysfunction, cognitive problems, or dysautonomia, during the course of their condition.
3. Large-scale studies addressing poorly studied NMS, such as pain and anxiety, are required as these are treatable issues in PD.
4. Early identification of NMS may have therapeutic implications, especially as some NMS may respond to dopaminergic drugs. In a 2-year follow-up study of a DNPD population, Erro *et al.*^106^ were the first to show that indeed specific NMS such as depression and concentration improved following dopaminergic treatment. Therefore, documentation of NMS in DNPD state could allow us to document how NMS respond to dopaminergic and non-dopaminergic treatment. Also, such a systematic documentation allows PD specialists to map the natural history of NMS in a comprehensive manner.
5. Regarding NMS, DNPD represents a unique and important patient group and allows one also to examine the clinical expression of the Braak staging of PD,^107^ in particular, expression of specific NMS. It also suggests that specific NMS may dominate the clinical picture of PD, suggesting specific subtypes within the NMS-dominant cluster of DNPD, as suggested by Erro *et al.*^21^ The natural history of such NMS-dominant subtypes are unclear and, from a pathophysiological point of view, these NMS-dominant subtypes confirm the differential spread of the pathophysiological process via a limbic or a brainstem route as has been documented by Jellinger.^108^ Clinically, identification of such subtypes may also have implications on treatment as ‘subtype-specific’ treatment packages may need to be developed. The review data also confirm the growing observation that assessment of NMS using validated clinical ‘holistic’ tools should be an integral part of clinical assessment in PD, supplementing motor assessment.

## Figures and Tables

**Table 1:** Characteristics of papers included in the review

*Year of publication*	
Range	1987–2015
	
*Number of publications per decade*	
Until 1990	6
1991–2000	14
2001–2010	19
2011–2015	55
	
*Number of DNPD patients per publication*	
Range	8–428
Mean (s.d.)	83.8 (96.0)
Median	45
	
*Number of publications studying…*	
Holistic profile	13
Autonomic domain	19
Neuropsychiatric domain	30
Sensory domain	7
Fatigue domain	3
Gastrointestinal domain	6
Sleep domain	16

Abbreviation: DNPD, drug-naive Parkinson’s disease.
